# Monitoring Seismic Velocity Changes Across the San Jacinto Fault Using Train‐Generated Seismic Tremors

**DOI:** 10.1029/2022GL098509

**Published:** 2022-09-30

**Authors:** Y. Sheng, A. Mordret, K. Sager, F. Brenguier, P. Boué, B. Rousset, F. Vernon, Q. Higueret, Y. Ben‐Zion

**Affiliations:** ^1^ University Grenoble Alpes University Savoie Mont Blanc CNRS IRD University Gustave Eiffel Grenoble France; ^2^ Department of Earth, Environmental and Planetary Sciences Brown University Providence RI USA; ^3^ Institut Terre et Environnement de Strasbourg Université de Strasbourg Strasbourg France; ^4^ Institute of Geophysics and Planetary Physics University of California, San Diego La Jolla CA USA; ^5^ Department of Earth Sciences and Southern California Earthquake Center University of Southern California Los Angeles CA USA

**Keywords:** long‐base seismic interferometry, train‐generated seismic energy, body‐wave correlation functions, Anza seismic gap, hidden slow‐slip event

## Abstract

Microseismic noise has been used for seismic velocity monitoring. However, such signals are dominated by low‐frequency surface waves that are not ideal for detecting changes associated with small tectonic processes. Here we show that it is possible to extract stable, high‐frequency body waves using seismic tremors generated by freight trains. Such body waves allow us to focus on small velocity perturbations in the crust with high spatial resolution. We report on 10 years of seismic velocity temporal changes at the San Jacinto Fault. We observe and map a two‐month‐long episode of velocity changes with complex spatial distribution and interpret the velocity perturbation as produced by a previously undocumented slow‐slip event. We verify the hypothesis through numerical simulations and locate this event along a fault segment believed to be locked. Such a slow‐slip event stresses its surroundings and may trigger a major earthquake on a fault section approaching failure.

## Key Points

- We extract stable station‐station body‐wave correlation functions using seismic energy generated by freight trains
- We perform 10‐year seismic velocity monitoring around the San Jacinto Fault and observe a 2‐month‐long velocity perturbation in 2014
- We interpret this velocity change as caused by a previously unreported slow‐slip event at the southern edge of the Anza seismic gap

## 1 Introduction

Fault zones release elastic energy stored in the Earth's crust by tectonic stresses in different ways. The energy release can happen rapidly and dramatically during earthquakes or gradually and aseismically during slow‐slip events (SSEs) (Kato & Ben‐Zion, 2021). SSEs that occur along active faults can represent a large portion of the slip budget (Peng & Gomberg, 2010), and their occurrence has also been recognized as a potential driving mechanism for major earthquakes (Kato & Ben‐Zion, 2021; Radiguet et al., 2016). Despite the substantial improvements in geodetic observations, noise levels prevent the detection of small SSEs, impeding the ability to describe the complete spectrum of transient slips and their roles in earthquake cycles.

Seismic velocities are sensitive to perturbations associated with earthquakes, such as damage caused by severe shaking or post‐seismic deformation (Brenguier et al., 2008). Velocity changes manifest as travel‐time variations among repeating signals, and their monitoring to provide a proxy for perturbations preceding large earthquakes remains a long‐sought‐after goal in seismology (Niu et al., 2008). The investigation of temporal changes of seismic velocity at crustal depths is hindered by the requirement for powerful and recurrent seismic sources that act over years. Low‐frequency ocean‐generated surface waves (0.1–1.0 Hz) have been used to study the responses of the Earth's crust to earthquakes (Brenguier et al., 2008) and volcanoes (Brenguier et al., 2014). However, the non‐localized sensitivity of long‐period surface waves limits the detection of strain transients associated with small deformations and the location of the origins of the related velocity perturbations.

**Figure 1:**
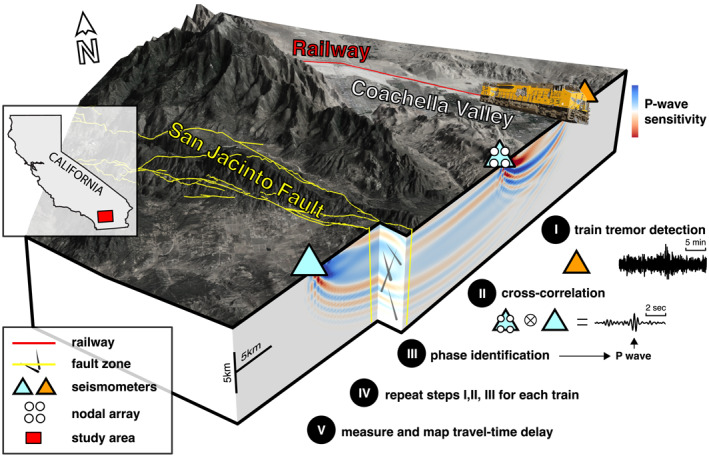
Schematic illustration of the location and layout of the study site and the data processing. Station–station correlation functions are repeatedly constructed using the seismic energy generated by freight trains. The compressional seismic body waves (*P*‐waves) are used in the correlation functions to monitor the San Jacinto Fault Zone continuously. The *P*‐wave sensitivity indicates where perturbations at depth can change the arrival times of interest. Increasing (decreasing) the velocity in the blue (red) zones leads to earlier arrivals of the compressional waves in the correlation functions. The main processing steps are explained in detail in the main text.

In this study, we apply a novel approach referred to as long‐base seismic interferometry, which uses correlations of anthropogenic seismic sources (e.g., freight trains) between sensors to retrieve compressional seismic body waves (*P*‐waves) (Brenguier et al., 2019). Instead of using low‐frequency surface waves or scattered waves to measure time‐lapse travel‐time perturbations (*dt*), we use high‐frequency ballistic *P*‐waves (>1 Hz) that travel down to a few kilometers in depth. These signals provide high sensitivity to velocity perturbations along the propagation path (sensitivity kernel in Figure 1) and reduced sensitivity to near‐surface environmental processes (Clements & Denolle, 2018), enabling a close monitoring of fault zone perturbations.

## 2 Analyses and Observations

We focus on the Anza seismic gap, a 20‐km section of the San Jacinto Fault in Southern California, USA. This seismic gap is bounded by two seismically active regions: the Hot Springs area to the north and the Trifurcation area to the south. The observed high strain rates (Johnson et al., 1994) and the absence of seismicity suggest that the Anza section is locked and can potentially host a magnitude 6.5+ earthquake (Sanders & Kanamori, 1984). Paleoseismic studies have estimated the recurrence interval of large earthquakes to be 254 ± 120 years (Rockwell et al., 2015) and indicated that the fault segment near Anza has not ruptured for more than 200 years. This area is densely equipped with geophysical instruments, making it an ideal natural laboratory for seismological research.

**Figure 2:**
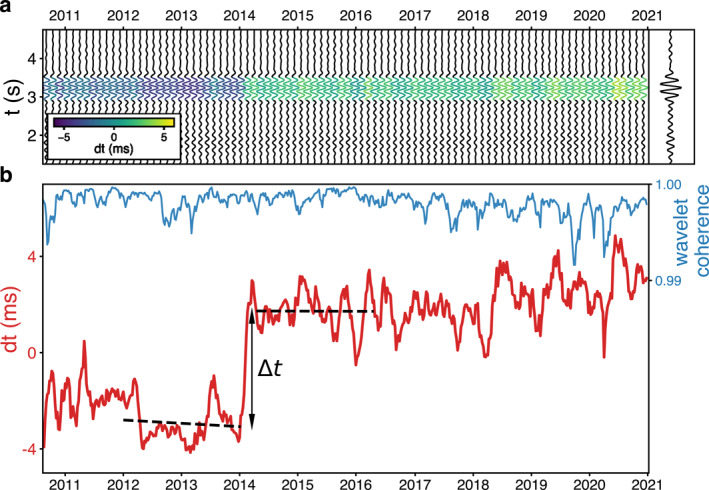
Long‐term travel‐time perturbations for the station pair II.PFO–AZ.FRD. (a) Weekly stacked correlation functions with 2‐month smoothing. The correlations are plotted every 6 weeks for visualization, and the long‐term average is shown on the right. The color represents the *dt* measurements relative to the long‐term average. (b) Ten‐year *dt* measurements and wavelet coherence. The black dashed lines show the linear regressions before and after the travel‐time offset in early 2014.

Train tremors excited in the nearby Coachella Valley have previously been considered a nuisance (Inbal et al., 2018); following recent studies (Brenguier et al., 2019; Pinzon‐Rincon et al., 2021), we turn train tremors into a valuable source of information. The Piñon Flat Observatory is used as the anchor site, where permanent seismic stations are accompanied by a temporary nodal array (network code 9K) for tracking moving trains and examining the feasibility of retrieving body waves in correlation functions. The temporary array was in operation for about 20 days in 2018. The main processing steps are illustrated schematically in Figure 1. First, the time intervals when freight trains pass by the study area are defined. Seismic interferometry is then performed by focusing on the train tremors to construct station–station correlation functions containing high‐frequency *P*‐waves. Finally, the long‐term travel‐time perturbations of the reconstructed *P*‐waves are estimated. Only the vertical component is used in this study. The detailed procedures are described further in the Supporting Information. The obtained station–station correlation functions show stable *P*‐wave energy (Figure S5 in Supporting Information S1) suitable for studying seismic velocity changes. Figure 2 presents an example data set obtained from station pair II.PFO–AZ.FRD.

**Figure 3:**
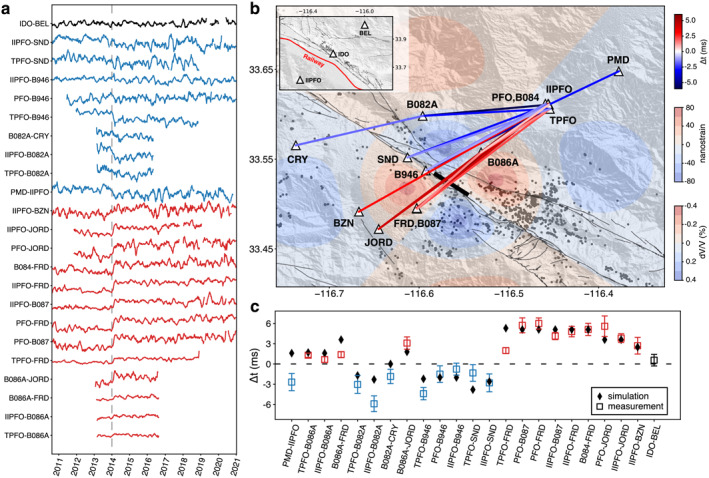
Travel‐time shift measurements. (a) Long‐term *dt* measurements for all of the considered station pairs. Pairs with positive (negative) *∆t* are shown in red (blue). The reference station pair IDO–BEL is shown in black. (b) Map of the station pairing for the measured *∆t*. The background colors indicate the volumetric strain at 1 km in depth from the preferred dislocation model (Table 1), with dilatational (compressional) strain in red (blue). The relative velocity change dV/V is calculated from the volumetric strain (Text S2 in Supporting Information S1). The thick black line marks the surface projection of the dislocation, and local earthquakes (Ross et al., 2019) during the 2‐month velocity‐change period are shown as black dots. The inset in the top left corner shows station II.PFO, CI.IDO, and CI.BEL. (c) Measured and simulated *∆t* values, using the same colors as in (a).

The analysis indicates a permanent velocity change in 2014 that lasted about two months. Such a velocity change differs from either coseismic velocity drop followed by a long‐term velocity recovery (Brenguier et al., 2008), or environmental perturbations typically presenting a periodic pattern (Clements & Denolle, 2018). Intriguingly, some station pairs show positive travel‐time shifts corresponding to velocity drops, while others are negative, representing velocity increases (Figure 3). We fit a step function in the least‐square sense for each pair of stations to quantify the travel‐time change offset (*∆t*) and its uncertainty in early 2014. Figure 2b illustrates the estimation for station pair II.PFO–AZ.FRD, and Figure 3c presents the results for every station pair.

There are no reports of changes to the railway system during this period. Theoretically, source changes lead to the same polarity changes of *∆t* for all station pairs, which is not observed. To further demonstrate that train‐related changes do not induce the observed travel‐time offset, the travel‐time perturbation is also measured on the station pair CI.IDO−CI.BEL, following the same procedure. These stations (Figure 3b and Figure S2 in Supporting Information S1) are in the vicinity of the Joshua Tree National Park, on the other side of the railway from the San Jacinto Fault zone, more than 40 km away from the Anza seismic gap. No evident *∆t* was observed between these two stations in early 2014 (Figure 3). We, therefore, argue that the travel‐time offsets are related to genuine seismic velocity changes near the Anza area. Environmental variations, such as groundwater level changes, can also alter seismic velocity (Clements & Denolle, 2018) in shallow structures; however, no significant precipitation was reported in the Anza region. In addition, body‐wave correlation functions are less susceptible to the perturbations near the Earth's surface, suggesting the possible cause of the velocity variation occurs at depth.

## 3 Possible Interpretation

The spatial distribution of *∆t* suggests a complicated pattern of velocity changes. Opening and closing voids or cracks can modulate the speed of compressional waves and yield either velocity decreases or increases (Walsh, 1965). Previous studies have argued for the existence of deep aseismic slip at the Anza seismic gap (Inbal et al., 2017; Wdowinski, 2009) and post‐seismic slip at nearby seismically active regions (Shaddox et al., 2021). Aseismic transients are also predicted at the bottom of the seismogenic zone in earthquake cycle simulations, with a proposed transition of heterogeneous frictional properties below the locking depth provided to explain the deep extent of the seismicity (Jiang & Fialko, 2016).

We search for a simple dislocation model to explain our velocity‐change observations and assess if they could have been caused by an aseismic slip. We use the Coulomb software (Lin & Stein, 2004) to simulate volumetric strain from an aseismic transient and convert the strain to seismic velocity change (dV/V) through depth‐dependant velocity‐stress sensitivity (Text S2 in Supporting Information S1). The modeled dV/V is then converted to travel‐time differences using full‐waveform simulations of correlation functions (Sager et al., 2022) with and without velocity perturbations (Text S2 in Supporting Information S1). The travel‐time differences measured from the simulated correlation functions are compared with the observations to adjust the dislocation model. To simplify the simulation, we assume a pure vertical slip patch rupturing along strike and fix the aspect ratio (length/width) as 2, which is reported as the medium value for SSEs at Parkfield (Tan & Marsan, 2020). The *∆t* polarity constrains the position of the slip patch close to the edge of the seismic gap. We perform a grid search over the center, the depth, the length, and the size of the slip. Each of the first three parameters varies at a 2‐km interval, and the slip size is chosen from 0.1, 0.2 to 0.4 m. The weighted summation of the misfit between simulations and observations is used to choose the ultimate model, with the inverse values of *∆t* uncertainties used as the weights. The simulated *∆t* values from the preferred model (Table 1) are presented against the observations in Figure 3c.

**Table 1:** Preferred Dislocation Parameters

Starting latitude	Starting longitude	Ending latitude	Ending longitude	Depth (km)	Right lateral slip (cm)	Dip (°)	Rake (°)
33.5113	−116.5470	33.5330	−116.5814	6–8	20	90	180

**Figure 4:**
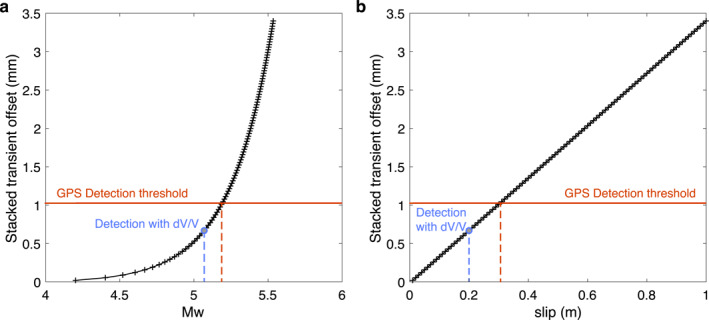
Minimum detection threshold with the current GPS network. Predicted GPS stacked offsets for a slip on the dislocation found with the *dt* analysis as a function of magnitude (a) and slip (b). The red lines indicate the detection thresholds with the current GPS network. The blues dots indicate the estimated magnitude and slip from the *dt* analysis.

Given the simplified velocity model, the assumed uniform elastic properties in the strain simulation, and the considerable uncertainty of the stress‐velocity sensitivity, finding the best dislocation model is nontrivial. Nevertheless, the simulated travel‐time changes from the preferred dislocation model fit the observations reasonably well in both polarity and amplitude (Figure 3c). The modeled slip is 0.2 m, and the equivalent moment magnitude is 5.1. This moment magnitude is slightly below the Global Positioning System detection threshold, estimated to be 5.2 (Figure 4; Text S3 in Supporting Information S1). We do not observe clear signals on strainmeters close to the fault. However, after the corrections for offsets, tidal strains, barometric pressure variations, and long‐term borehole trends, month‐long strain fluctuations still present and dominate the strain measurements (Figure S8 in Supporting Information S1). The simulated volumetric strains at nearby strainmeters are of the order of 10 nanostrain, much smaller than the fluctuations. Such small magnitudes possibly explain the lack of related observations from standard geophysical methods.

## 4 Discussion and Conclusion

**Figure 5:**
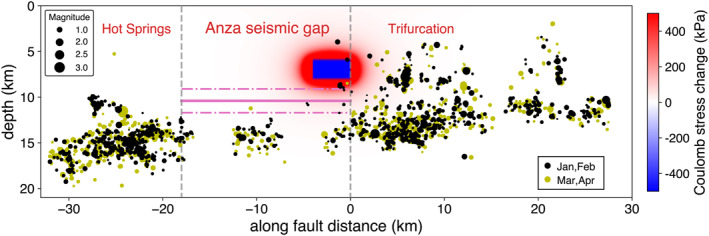
The along‐fault cross‐section of Coulomb stress changes from the dislocation slip model and the earthquakes within 3 km of the fault zone. Earthquakes in January and February 2014 are shown in black, and those in March and April 2014 are yellow. The number of earthquakes surrounding the dislocation dropped significantly after the slip (Figure S9A in Supporting Information S1). The solid magenta line represents the geodetic locking depth (Lindsey et al., 2014), and the dashed lines mark its uncertainty.

Unlike subduction SSEs accompanied by abundant seismic activity (Beroza & Ide, 2011), only a few earthquakes occurred in the vicinity of the inverted SSE during the 2‐month period (Figure 5). The majority of these earthquakes appear as a swarm at a depth of 8.8 km. The general quiescence around the slipped patch suggests that this earthquake swarm was likely triggered. The lack of seismicity in the vicinity of the SSE is not surprising, given the locked nature of the fault segment (Sanders & Kanamori, 1984). Inbal et al. (2017) also reported similar findings for deep aseismic transients in the same region. SSEs at strike‐slip faults are often not accompanied by tectonic tremors (Rousset et al., 2016), probably due to the different rheology and conditions compared to subduction zones. Inbal et al. (2017) observed an increasing seismicity rate in the Hot Springs and Trifurcation region associated with deep aseismic transients. They explained the rate increase as the result of creep‐induced fault loading. We also observe an elevated seismicity rate at the beginning of 2014, especially in the Trifurcation area (Figure S9B in Supporting Information S1); however, whether this change is related to the modeled aseismic slip requires further investigations.

The preferred dislocation model is at the bottom of the locked fault section (Lindsey et al., 2014), consistent with a transition zone between seismogenic velocity‐weakening and deeper velocity‐strengthening regions. Such mechanical heterogeneity can reflect a complex structure (Jiang & Fialko, 2016) or spatially variable pore pressure suggested by fluid‐driven swarms (Ross & Cochran, 2021). The modeled SSE is also located at the southern end of the seismic gap, at the transition to the zone with high seismic activity, indicating possible lateral variations of mechanical properties. The modeled SSE had a stress drop of a few hundred kPa, in the upper range for SSEs (Bartlow et al., 2014). The resulting positive Coulomb stress change in the seismic gap is significant enough to trigger a large earthquake (Radiguet et al., 2016). It is worth noting that we do not observe appreciable travel‐time perturbations associated with local moderate‐sized earthquakes (Ross et al., 2019), which are accompanied by aseismic slip revealed by strainmeter data and near‐repeating earthquakes (Shaddox et al., 2021). Simulations for the M4.7 March 2013 Anza Earthquake (Figure S10 in Supporting Information S1) show that the induced strain has smaller amplitudes than the SSE and negligible travel‐time changes. The strongly affected region is also further away from the sensitivity zone of the reconstructed interferometric body waves.

We observe a complicated pattern of seismic velocity change and interpret it as a result of an SSE at depth. Despite other possible explanations, the source of change should not be shallow, given the body‐wave sensitivity. Detecting hidden fault movements significantly improves the understanding of the interaction between aseismic and seismic slips, a necessary step to characterize large earthquakes' preparation phase. The presented methodology and results rely on freight trains as seismic sources; however, other opportune sources, for example, car/truck traffic and industrial activities, could also be used after careful examinations of source signatures. Using anthropogenic seismic signals opens up new possibilities for monitoring geological targets of interest, such as fault systems, volcanoes, and geothermal or carbon storage reservoirs.

## Supporting information

Supporting Information S1

## Data Availability

All the seismic waveform data used can be accessed through IRIS. The networks include the ANZA Seismic Network (https://doi.org/10.7914/SN/AZ), the Southern California Seismic Network (https://doi.org/10.7914/SN/CI), the 2018 FaultScan dense array network (https://doi.org/10.7914/SN/9K_2018), and the Plate Boundary Observatory Borehole Seismic Network (no DOI available at present, but the information could be accessed through https://www.fdsn.org/networks/detail/PB/). The processed borehole strainmeter data (https://www.unavco.org/data/strain-seismic/bsm-data/bsm-data.html) and Global Positioning System data (https://www.unavco.org/data/gps-gnss/gps-gnss.html) were obtained from UNAVCO (https://www.unavco.org). We use pycorr software package (https://doi.org/10.5281/zenodo.6793401) to download the continuous seismic data and perform seismic interferometry. The dt measurement is calculated using the wavelet method (https://doi.org/10.5281/zenodo.4783514); the Coulomb 3 software is used for the strain simulation (https://www.usgs.gov/node/279387); the full‐waveform simulation of the correlation wavefield is developed based on the Salvus package (https://mondaic.com), and the script for this study can be found at https://doi.org/10.5281/zenodo.6792393.
