# Supplementary material for: Monitoring Seismic Velocity Changes Across the San Jacinto Fault Using Train‐Generated Seismic Tremors
Source: Geophys Res Lett. 2022 Sep 30;49(19):e2022GL098509. doi: 10.1029/2022GL098509 (PMC9786557; doi:10.1029/2022GL098509)
Supplement: Supplementary file 1 — Supporting Information S1 [file GRL-49-e2022GL098509-s001.pdf]

AGU PUBLICATIONS

Geophysical Research Letters

Supporting Information for

Monitoring seismic velocity changes across the San Jacinto fault using train-generated seismic tremors

Yixiao Sheng[1], Aurélien Mordret[1], Korbinian Sager[2], Florent Brenguier[1], Pierre Boué[1], Baptiste Rousset[3], Frank Vernon[4] and Yehuda Ben-Zion[5], Q. Higueret[1]

1 Univ. Grenoble Alpes, Univ. Savoie Mont Blanc, CNRS, IRD, Univ. Gustave Eiffel, ISTerre, 38000 Grenoble, France

2 Department of Earth, Environmental and Planetary Sciences, Brown University, Providence, RI, USA

3 Institut Terre et Environnement de Strasbourg, Université de Strasbourg, France

4 Institute of Geophysics and Planetary Physics, University of California, San Diego, La Jolla, CA, USA

5 Department of Earth Sciences and Southern California Earthquake Center, University of Southern California, Los Angeles, CA, USA

Contents of this file

Text S1 to S3
Figures S1 to S10

Introduction

This supporting information adds detailed descriptions of the analyses, which include three parts. Text S1 introduces the processing of seismic data. Text S2 describes the numerical simulations that are used to support the hypothesis of velocity change triggered by aseismic slip. Text S3 explains the processing procedures for GPS signals. Figures provide additional support to both manuscript and the supporting texts.

Text S1: Train detection, seismic interferometry, and travel-time delay measurement

S1.1. Train detection.

Identifying train-generated seismic energy directly for stations near the Anza seismic gap is challenging, given the long distance to the railroad (~35 km) and the seismically active environment. Beamforming results (Figure. S1) from the nodal array at Piñon Flat Observatory (Figure. S2) suggest the train-generated energy shows a peak in the study area as the trains pass by seismic station CI.IDO, which is 6 km from the railroad. Station CI.IDO was therefore used as a reference to time the train passes. The procedure is illustrated in Figure. S3 and can be described as follows.

First, a band-pass filter between 3 Hz and 5 Hz was applied to the raw seismogram to reveal the train energy. Then, 2-min running window smoothing was applied to the seismogram envelope to suppress the local earthquakes. The peaks from the smoothed envelope were picked, with the requirement that each peak had an amplitude >2.5 times the median of the trace and a duration >3 min. A 12-min time window centered around each peak is defined as the span of each train. This time window is long enough to contain most of the train energy seen at station CI.IDO. The procedure was restricted to nighttime to avoid potential contamination from the local traffic noise during the day.

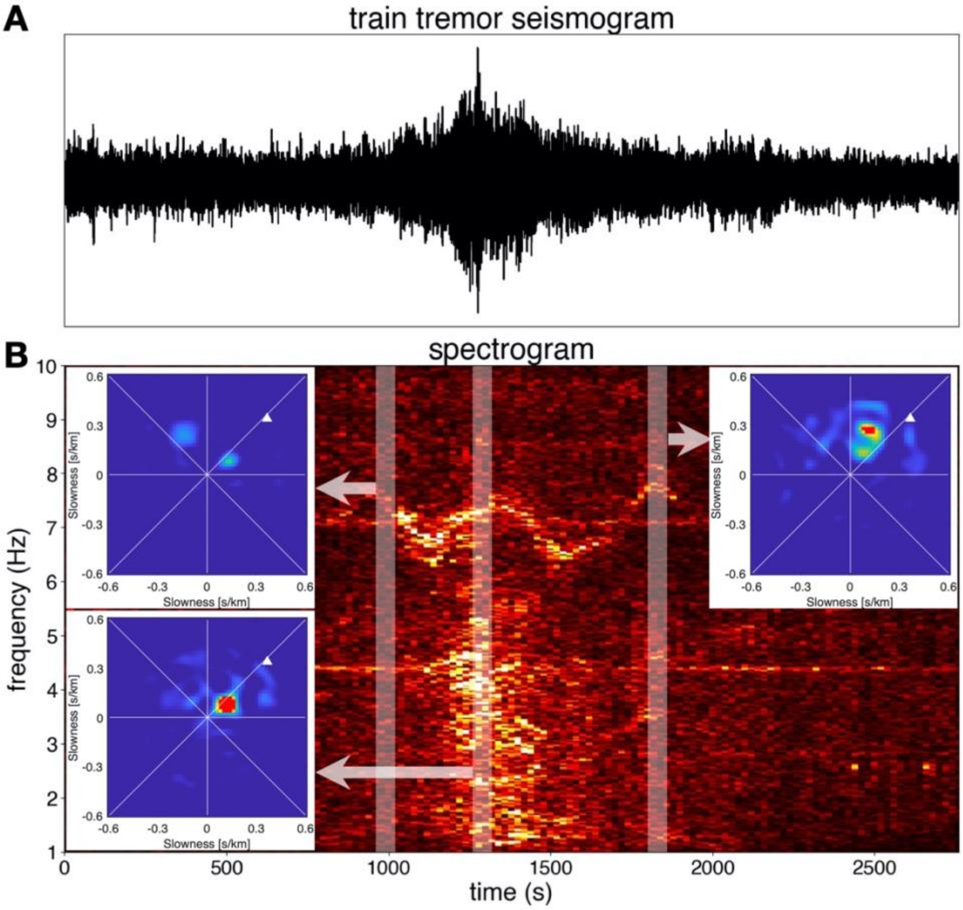

Figure S1. An example of train-generated seismic signals. (A) Seismogram recorded at station CI.IDO. (B) Spectrogram of (A), with three insets showing the 1-min beamforming results using the temporary nodal array at the Piñon Flat Observatory. The frequency range used in the beamforming was 2 Hz to 6 Hz. The white triangles in the insets mark the azimuth of station CI.IDO to the nodal array.

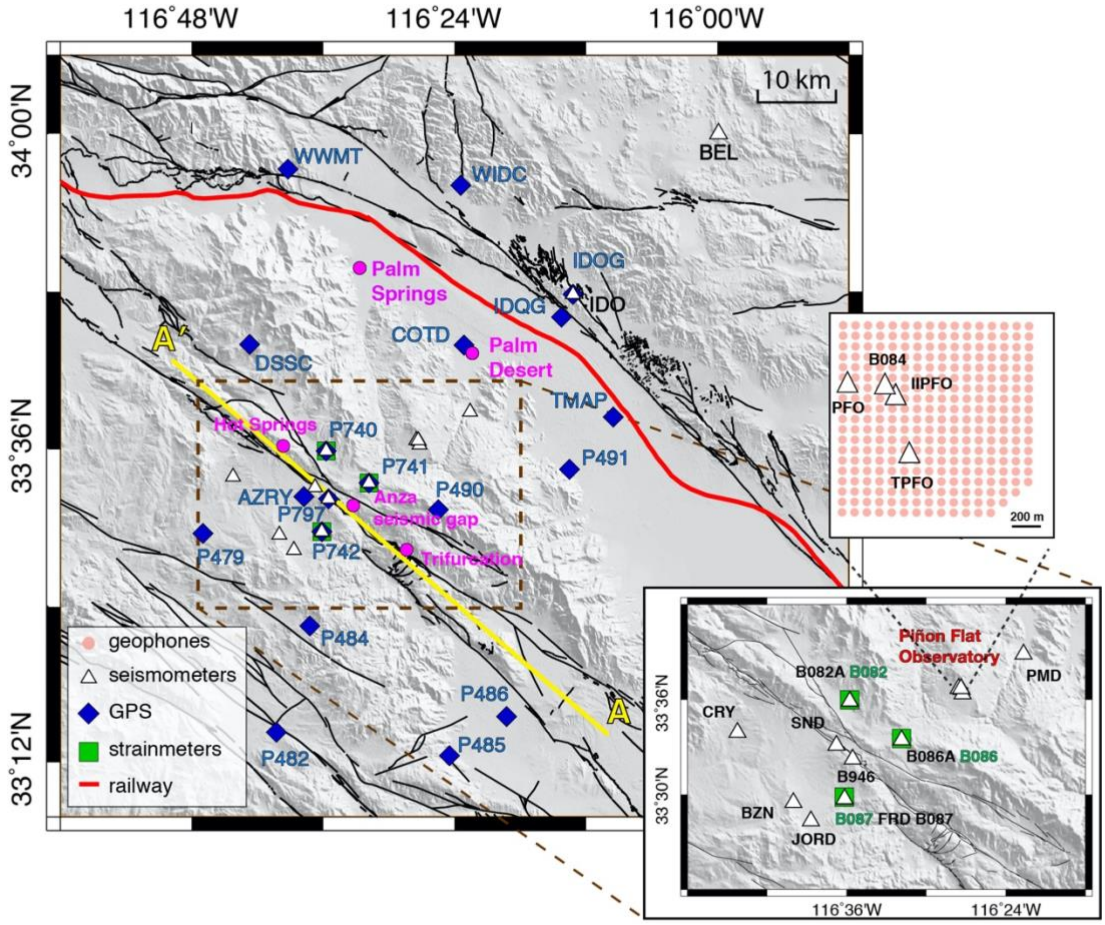

Figure S2. Map of the study area with all the geophysical instrument networks shown.

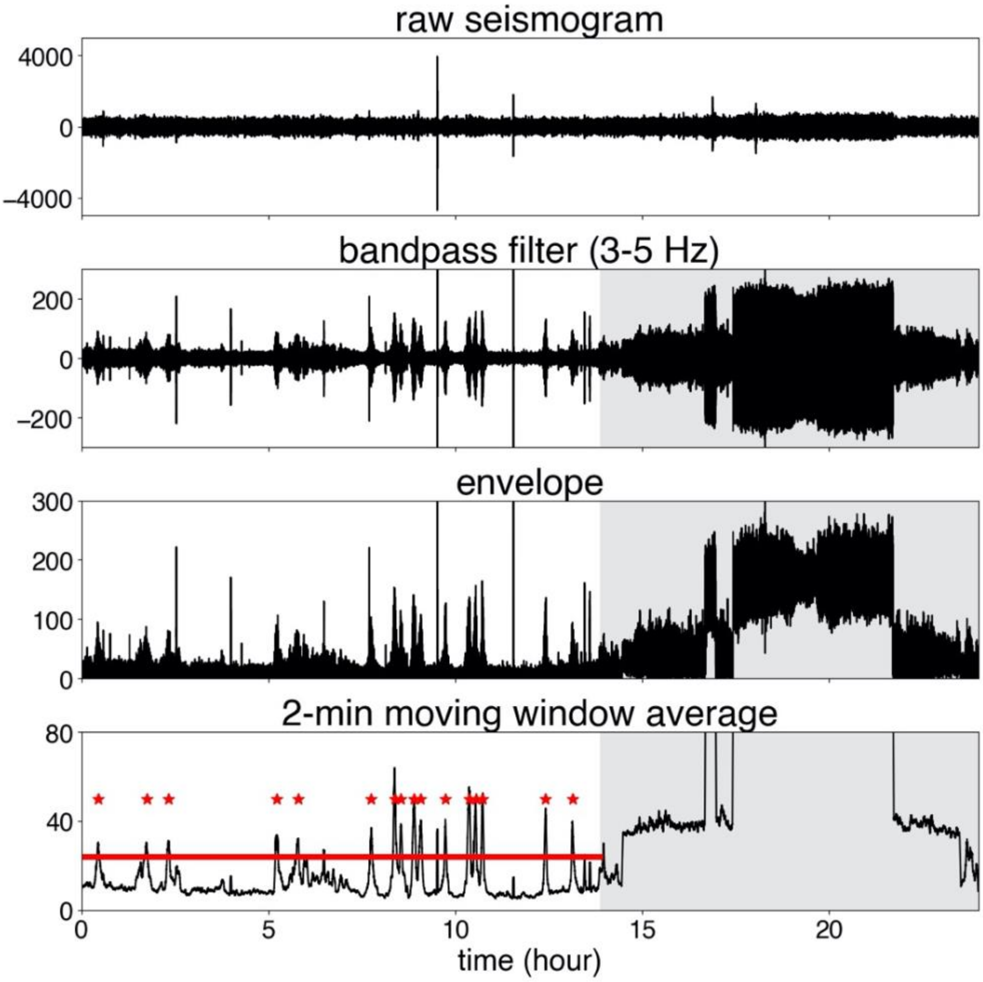

Figure S3. Detection procedures of train tremors on a single station. The red line in the bottom panel indicates the detection threshold, and the red stars mark the train tremors detected. The detection is excluded during local daytime, as indicated by the grey shading.

S1.2. Seismic interferometry: array-station correlation

Once the trains catalogue had been compiled, seismic interferometry was performed, focusing on the train-generated seismic waves. Most of the permanent seismic stations in the study area had broadband channels with a sampling rate of 40 Hz. An anti-aliasing low-pass filter was applied for stations with higher sampling rates, with a cut-off frequency at 18 Hz and down-sampling of the seismogram to 40 Hz for further estimation. Instrument responses were not removed to avoid potential bias from deconvolution. For each train, cross-coherence was carried out over the entire 12-min window. Before moving towards station-station correlation, the array-station correlation was tested. Fifty-meter distance-bin stacking was applied to improve the signal-to-noise ratio (SNR). In the examples shown in Figure. S4, a clear body-wave moveout can be seen, with an apparent velocity of 6 km/s. Compared to the correlations of continuous seismic recordings, the exclusive correlation of train tremors shows significant improvements for the SNR of the correlation functions.

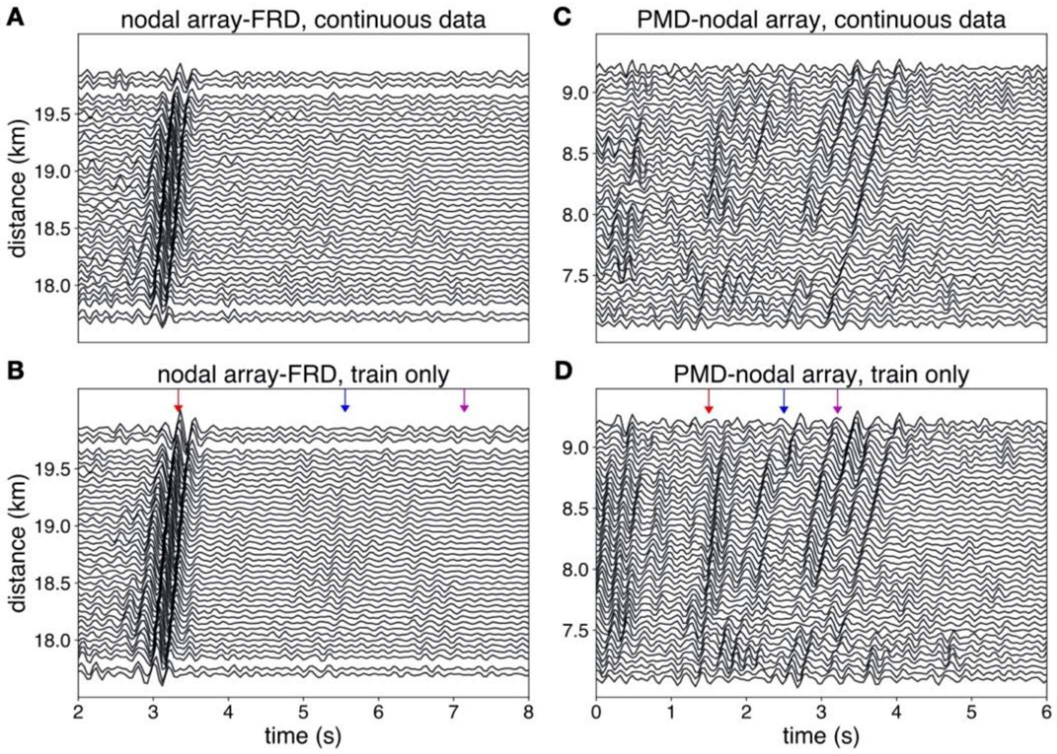

Figure S4. Comparisons between blindly stacked correlation functions from continuous data and selective correlation functions focused on train tremors. (A, B) Correlation functions from the nodal array to station AZ.FRD. (C, D) Correlation functions from station CI.PMD to the array. In each plot, a 50-m distance-bin stacking is applied, and the waveforms are bandpass filtered between 3 Hz and 8 Hz. Red, blue, and magenta arrows in (B, D) indicate P, S and surface waves.

S1.3. Seismic interferometry: station-station correlation

The successful application of array-station correlation further motivated the station-station correlation. Cross-talk of correlated sources, such as train signals generated from different locations, might introduce spurious arrivals and complicate the correlation functions (Ayala-Garcia et al., 2021). Moveout that appeared on the array-station correlation functions was used as a reference to identify the target P-wave (Figure. S5A). The dense nodal array is beneficial to identify the target P phase when the virtual wavefield is complicated. As local earthquakes can contaminate individual correlation functions, quality control was carried out. The SNR was first used as a criterion. For each correlation function, the SNR is defined as the ratio between the peak amplitude of the envelope of the P-wave and the peak amplitude in a 2-s-long window centred around the zero-time lag. This criterion works well for station pairs that do not suffer much from cross-talk, possibly because most of the train energy radiates from the stationary phase zone. For station pairs with strong acausal arrivals on the correlation functions, separation of good and biased correlation functions is nontrivial. Therefore, another selection criterion was introduced, as the phase synchrony, to determine the quality of each correlation function. Phase synchrony is defined as the average difference between the instantaneous phases of two compared time series. The long-term averaged correlation function was used as the reference, with the calculation of the phase synchrony focused on the targeting P-phase. One correlation function was kept for each station pair if both the SNR and the phase synchrony were in the top 30% of all correlation functions. We find that these criteria yield a reasonable number of correlation functions without adding too many constraints. The same selecting criteria were applied to all station pairs to be consistent. Figure. S5B presents the long-term stacked correlation functions displayed as a function of distance. P-wave moveout clearly emerges.

S1.4. Travel-time delay measurement

Weekly stacking was performed on the selected correlation functions, with the application of 2-month smoothing before measuring the travel-time perturbations. Each correlation function was compared with the long-term average (Figure. S5B). For each station pair, P-wave is manually identified and selected. A window of 0.5 second centring the P-wave is used for measuring travel time difference. The wavelet method was applied (Mao et al., 2020) to obtain the weighted average time shift between 4 Hz and 6 Hz, with the wavelet coherence as the weight. The 4 Hz to 6 Hz frequency band showed the highest coherence among all station pairs. We use the Morlet wavelet in the analysis.

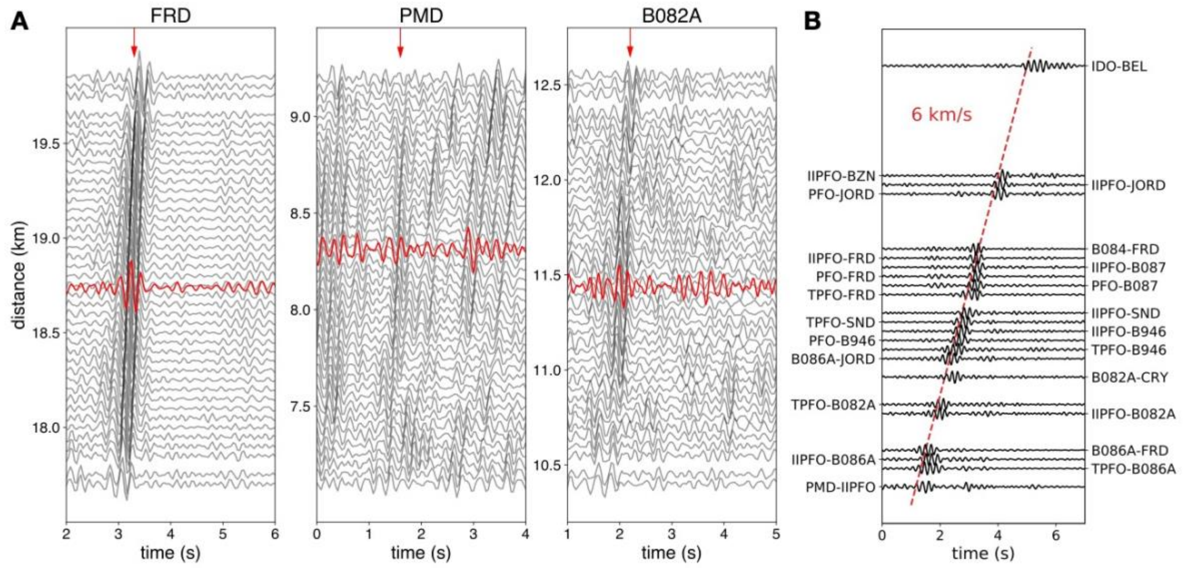

Figure S5. Correlation functions. (A) Examples of station-station correlation functions (red) from station II.PFO at the Piñon Flat Observatory to stations AZ.FRD, CI.PMD, and PB.B082A, accompanied by the array-station correlation functions (black). The station-station correlation functions were averaged from those obtained in 2018, and the array-station correlation functions were stacked in 50-m-distance bins. Red arrows indicate the P-wave. Note the complicated wavefields in the middle panel. (B) Long-term averaged reference correlation functions for all station pairs considered. These correlation functions are aligned with increasing distances, and some station pairs are shifted slightly upwards or downwards for visualization purposes. All the waveforms are bandpass filtered between 3 and 8 Hz.

Text S2: Model P-wave travel-time change from volumetric strain perturbations

S2.1. Transfer strain to relative velocity change

The volumetric strain (ε) is translated to the relative velocity perturbation (dV/V) through Equation (s1):

$$\frac{dV}{V} = -\eta K \varepsilon \qquad \text{(s1)},$$

where K is the bulk modulus, fixed as 50 GPa, and η is the velocity-stress sensitivity, which decays exponentially with increasing depth (Silver et al., 2007). As no in-situ measurement of η is available, values from 10^{-9} Pa^{-1} to 10^{-6} Pa^{-1} were used, as reported in previous studies (Yamamura et al., 2003), and a profile was formulated following Equation (s2):

$$\eta = 10^{-5-depth} \qquad \text{(s2)},$$

where the depth is in km.

Considering the extensive fault system in the study area, a large density of pore space and cracks was expected and was also supported by the observed fluid-driven swarms (Ross and Cochran, 2021). The relatively large η in the shallow layer is, therefore, reasonable.

S2.2. Modelling correlation functions

The modelled dV/V was then mapped to the travel-time differences using full-waveform simulations of correlation functions without and with velocity perturbations. To simulate correlation functions, we followed an approach introduced from helioseismology to terrestrial seismology (Tromp et al., 2010) and recently adapted to study compressional waves (Sager et al., 2022). Under certain conditions, correlation functions can be interpreted as inter-station Green's functions, i.e., the response of the medium due to an impulse source (Lobkis and Weaver, 2001). For this interpretation to hold, essentially, a homogeneous distribution of seismic sources is required. As trains are localised in space, Green's function retrieval principle was dropped, and synthetic correlation functions were modelled, where the specific distribution of seismic sources is accounted for. In addition, this approach allows computation of the spatial sensitivity patterns of the arrivals of interest to structural perturbations. In this study, a distributed source along the railway was used to approximate the trains, and the average 1D velocity model (Fang et al., 2016) was used as the base velocity. Two examples of simulated correlation functions and the corresponding sensitivity kernels are shown in Figure. S6. We refer the reader to a companion study (Sager et al., 2022) for more details.

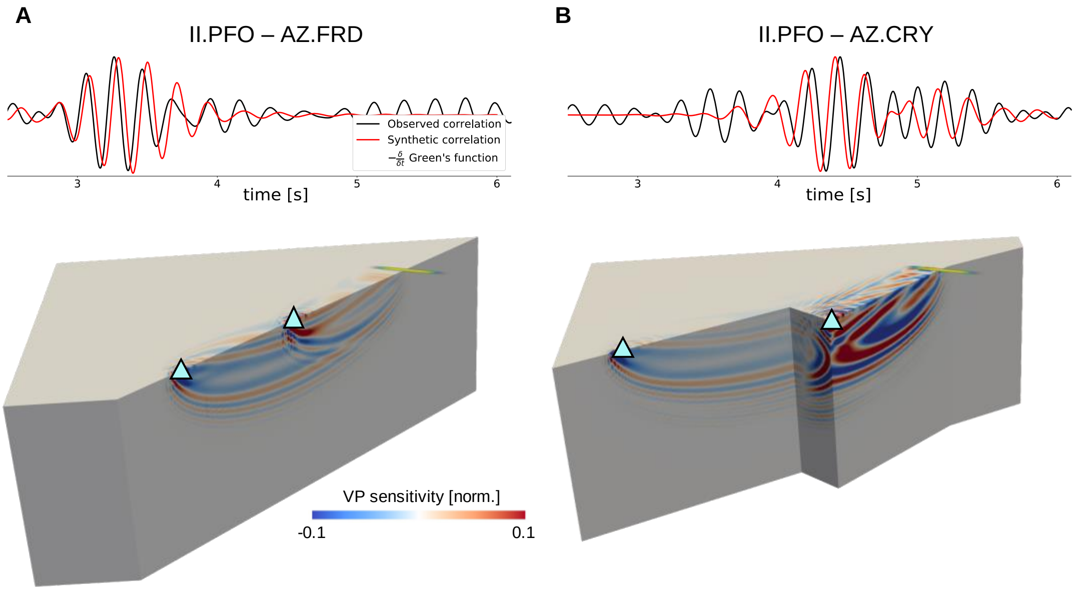

Figure S6. Examples of modelled correlation functions and P-wave sensitivity kernels. (A) station pairs II.PFO–AZ.FRD and (B) II.PFO–AZ.CRY. The simulated correlation functions match the observations well, except for the minor travel-time mismatch due to the simplified 1D velocity model.

Text S3: GPS analysis

S3.1. Processing of the GPS times series

The 30 GPS stations around the Anza gap of the San-Jacinto Fault used in this study were installed by the Plate Boundary Observatory (PBO; shown in Figure. S2). The GPS PBO level 2b combined solutions (Herring et al., 2016) were post-processed in two steps. In the first step, a trajectory model (Bevis and Brown, 2014) was used that comprised a linear term to account for the long-term tectonic loading, Heaviside functions, and logarithmic decreasing functions to correct for the coseismic and post-seismic signals associated with the 1999 Mw 7.1 Hector Mine earthquake and the 2010 Mw 7.2 El Mayor Cucapah earthquake. In the residuals, transient signals of seasonal loading origin can be observed, and 2-month-long transient signals with a static offset of up to 1 cm in August 2014 and January 2015. These two transient signals were not associated with slip on the San Jacinto Fault because the displacement was homogeneous at the analysed network scale, with a consistent eastward motion. To correct for these transient signals, another trajectory model was used in a second step, which included sine and cosine functions with periods of 1 year and six months to account for the seasonal signals, as well as half a cosine cycle with a duration of 30 days and 45 days to correct for the static transient offsets in 2014 and 2015, respectively.

S3.2. Stacking of the GPS time series

In the residual time series, no clear transient can be seen for the individual GPS time series in January and February 2014. To increase the SNR associated with the transient event observed in the *dt* time series, all GPS time series were stacked, each weighted by the predicted surface offset for 1 m of slip at the preferred dislocation found for the *dt* analysis (Rousset et al., 2019). Such weighting accounts for the correct polarity expected at each GPS site and gives more weight to the sites expected to produce larger static offsets. The resulting stacks for the East, North and combined East and North components are shown in Figure. S7. The stacks are compared to the slow-slip event surface deformation expected from the preferred slow-slip dislocation derived from the *dt* analysis. With the existing GPS network, such a transient surface deformation has an amplitude too small to emerge from the stacked GPS noise.

S3.3. Minimum moment detection with the GPS network

The minimum Mw that can be detected from the current GPS network for a slow-slip event on the Anza gap at the slip location found with the *dt* analysis was derived. We assumed that a transient event could be detected in the stacked GPS time series that combines the East and North components if it produces a stacked surface offset larger than three times the standard deviation of the GPS noise. With such a threshold, a transient slip event could be observed by the GPS network if it had a magnitude Mw >5.2 or a slip amplitude >31 cm for the 8 km^2 dislocation found in the *dt* study (Figure. 4). This GPS analysis enabled the validation of an upper bound for the moment released by the slow slip. Above this bound, the signal should have been captured by the GPS network.

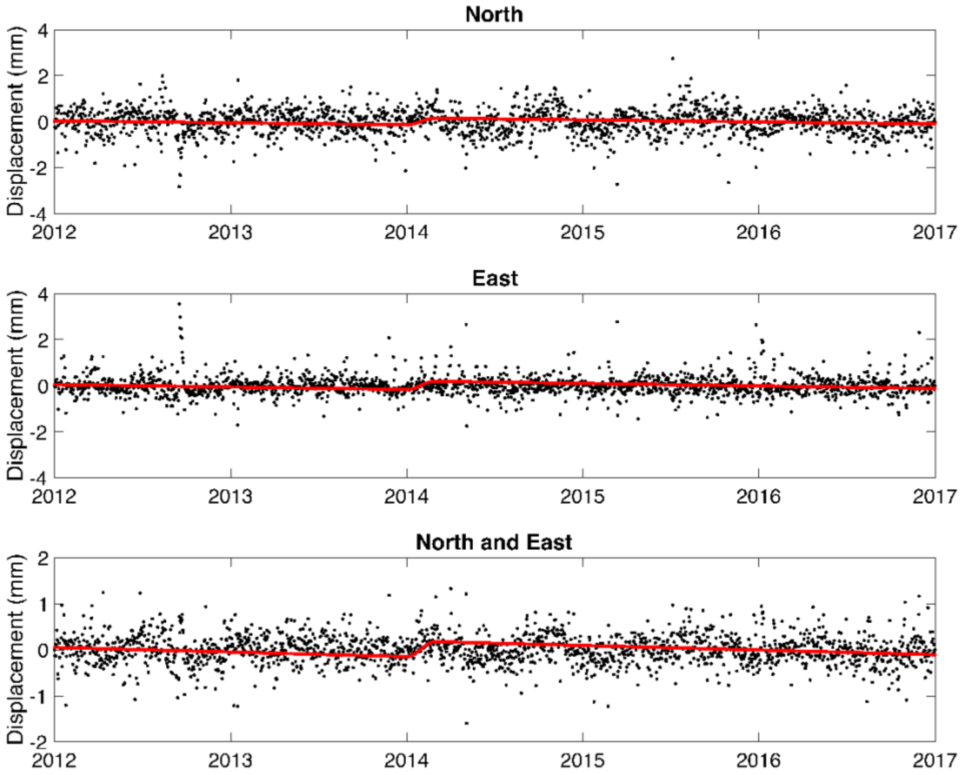

Figure S7. Stacked GPS time series and prediction from the slip dislocation. Weighted stacks of the GPS time series (black dots) and weighted stacks predicted by the slow-slip event dislocation identified through the *dt* observations (red lines). The top and middle panels correspond to the stacked time series for the East and North GPS components, respectively. The bottom panel shows the combined stacks.

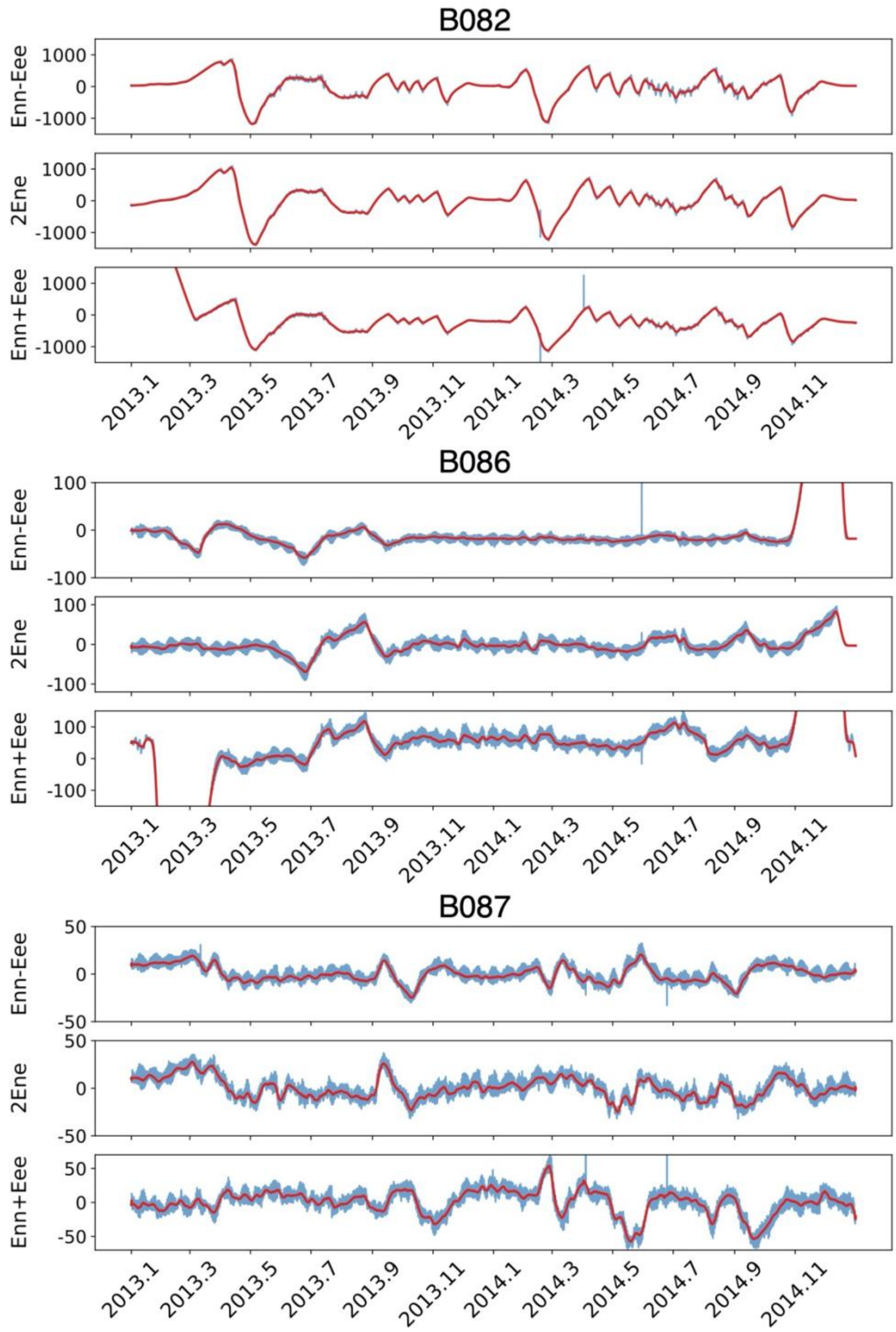

Figure S8. Strain data (in nanostrain) near the Anza seismic gap. The locations of the strainmeters are shown in the Figure. S2. The blue curves show the strain data after the corrections for offsets, tidal strains, barometric pressure variations, and long-term borehole trends and the red curves show the smoothed version to reveal the trend. The exceptionally large value at B082 is possibly due to a water pumping station close by (Inbal et al., 2017). Large fluctuations also present at the other two strainmeters.

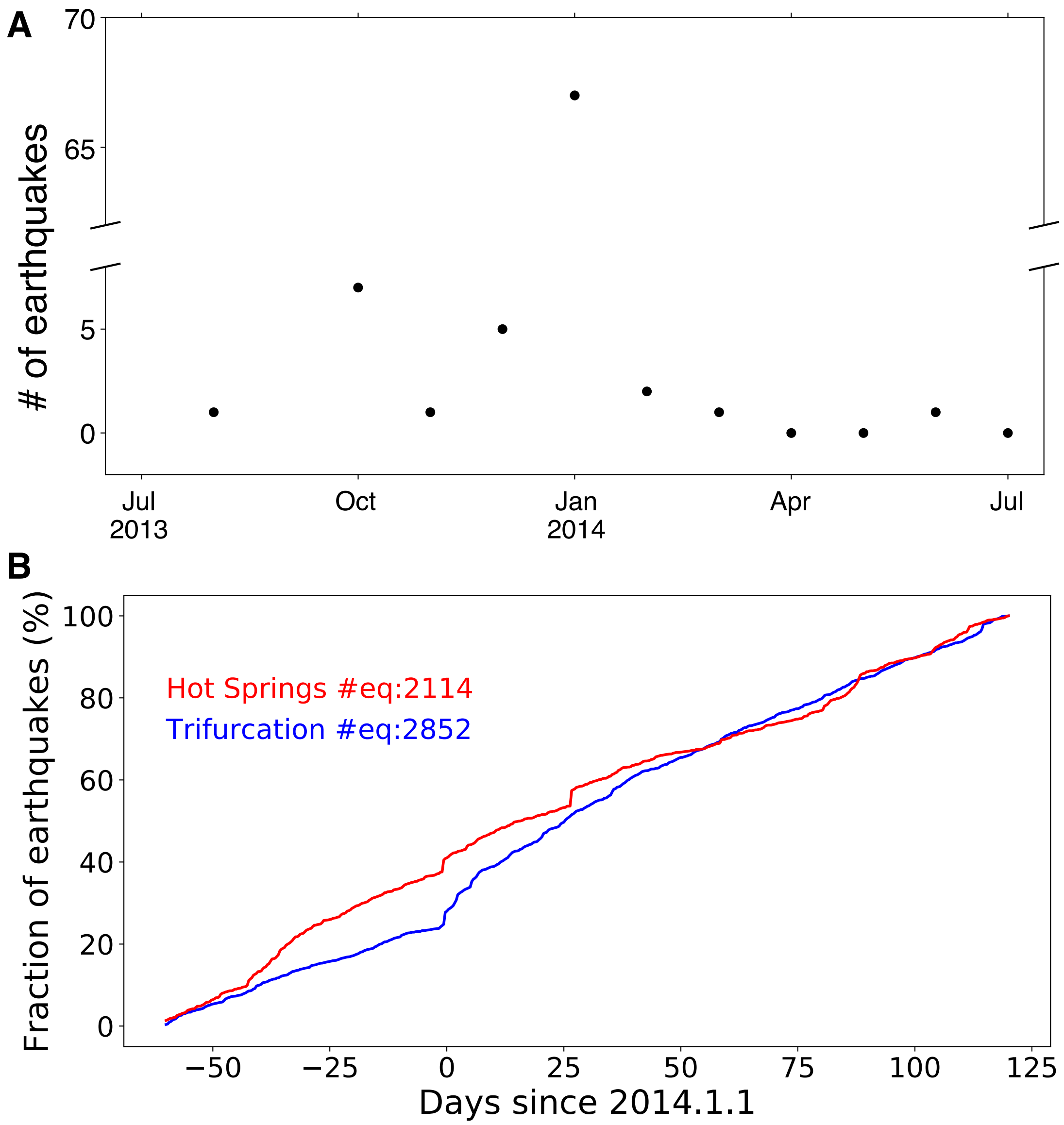

Figure S9. Background seismicity analysis. (A) Monthly count of earthquakes within 1 km of the inverted slip patch. January 2014 shows a clear peak, which suggested that those events were likely to have been triggered by the slow-slip event. (B) Cumulative event count as a function of time in the Hot Springs and Trifurcation areas. Earthquakes in the QTM catalog within 3 km to the A-A' cross-section (shown in Figure S2) are considered. Note the increasing seismicity rate at the beginning of 2014.

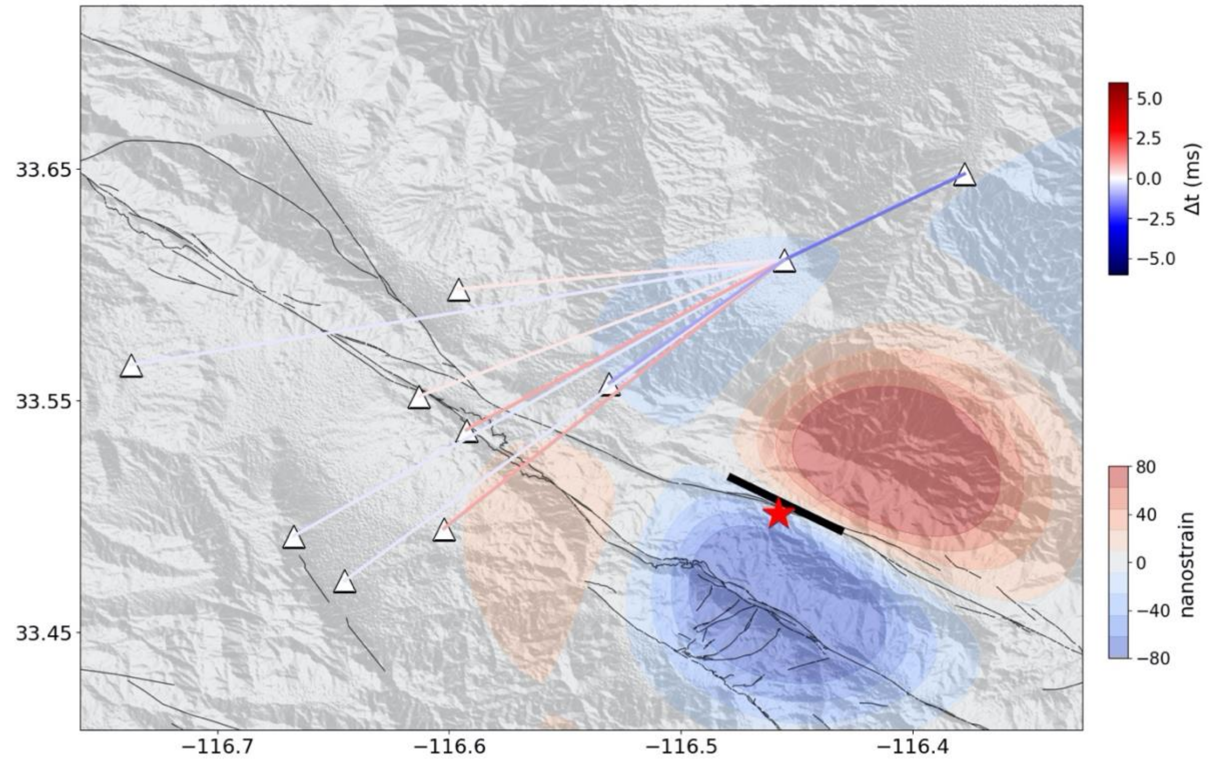

Figure S10. Simulations for the M4.7 March 2013 Anza Earthquake. Among the moderate-sized earthquakes that occurred in the Trifurcation area, this earthquake is the closest to the Anza seismic gap, while recorded by most of the seismic stations used in this study. The red star marks the event's epicentral location, and its focal mechanism is obtained from Southern California Seismic Network. The black thick line marks the surface projection of the earthquake afterslip reported in Shaddox et al. (2021). The background colors indicate the accumulated volumetric strain from both the earthquake and the afterslip at 1 km in depth. The strain amplitudes in our study area are much smaller compared to those in Figure. 3B in the vicinity of the seismic stations. The resulting travel-time changes are mostly smaller than 1 ms, which is difficult to be detected.
